# Using mobile phones to ensure that referred tuberculosis patients reach their treatment facilities: a call that makes a difference

**DOI:** 10.1186/s12913-017-2511-x

**Published:** 2017-08-22

**Authors:** Kimcheng Choun, Shanta Achanta, Balaji Naik, Jaya Prasad Tripathy, Sopheak Thai, Natalie Lorent, Kim Eam Khun, Johan van Griensven, Ajay M. V. Kumar, Rony Zachariah

**Affiliations:** 1 Sihanouk Hospital Centre of HOPE, Street 134, Sangkat Vealvong, Khan 7 Makara, Phnom Penh, Cambodia; 2 WHO country office for India, New Delhi, India; 3 School of Public Health, PGIMER, Chandigarh, India; 4 Institute of Tropical Medicine, Antwerp, Belgium; 5 National Tuberculosis Control Programme, Phnom Penh, Cambodia; 6 International Union Against Tuberculosis and Lung Disease, South-East Asia Regional Office, New Delhi, India; 7 Médecins Sans Frontiéres, Brussels Operational Centre (Operational research), Luxembourg city, Luxembourg

**Keywords:** Operational research, mHealth, Tracking, Referrals, SORT IT, LTCR

## Abstract

**Background:**

Over the last decade, the availability and use of mobile phones have grown exponentially globally and in Cambodia. In the Sihanouk Hospital Centre of Hope(SHCH) in Cambodia about half of all tuberculosis patients referred out to peripheral health facilities for TB treatment initiation or continuation were lost to contact after referral ranging from 19 to 69% between 2008 and 2013. To address this, we implemented a mobile phone-based patient tracking intervention. Here, we report the number and proportion of referred TB patients who could be contacted through a mobile phone and retained in care after the introduction of mobile phone tracking.

**Methods:**

A descriptive study involving follow-up of TB patients referred out from SHCH to peripheral health facilities during May–October 2014. Standard operating procedures were used to contact individual patients and/or health facilities using a mobile phone.

**Results:**

Among 109 TB patients referred to peripheral health facilities, 107(98%) had access to a mobile phone of whom, 103(97%) could be contacted directly while 5(2%) were contacted through their health care providers. A total of 108(99%) of 109 referred TB patients in intervention period were thus placed on TB treatment.

**Conclusions:**

This study provides preliminary, but promising evidence that using mobile phones was accompanied with improved retention of referred TB patients compared to historical cohorts. Given the limitations associated with historical controls, we need better designed studies with larger sample size to strengthen the evidence before national scale-up.

## Background

Technologies such as cellular (or mobile) phones when used for health related interventions constitute what are termed mHealth (mobile health) interventions. The World Health Organization (WHO) has defined mHealth as ‘medical and public health practice supported by mobile devices, such as mobile phones, personal digital assistants and other wireless devices [1].

Over the last decade, the availability and use of mobile phones have grown exponentially with over 4.5 billion mobile phone subscriptions now counted in the developing world - reaching about 90% of the world’s population [2]. In many places, people are now more likely to have access to a mobile phone than clean water, a bank account, or a source of electricity [3]. Cambodia, a low-income country in South-East Asia is no different, with 20 million mobile phone subscriptions for a population of 14 million. Mobile phones thus constitute a largely untapped resource which may provide an opportunity for improving health care in low and middle income countries (LMICs).

The Sihanouk Hospital Centre of Hope (SHCH) in Phnom Penh is a non-governmental hospital providing care free-of-charge for poor and vulnerable groups. It is one of the five tertiary health care facilities in the capital city of Cambodia. As care is free, large numbers of patients with presumptive tuberculosis (TB) seek care in the SHCH from across the country with considerable numbers being diagnosed with TB. About half of all such individuals are referred out to peripheral health facilities for TB treatment initiation or continuation - the latter includes those initiated on TB treatment at SHCH due to severe disease and once stabilized referred for treatment continuation. Since 2008, the hospital has recognized that a considerable proportion of such patients is ‘lost to contact after referral’ (LTCR) and it is not known whether these patients reached the peripheral health facilities and started treatment or not (Table 1).

**Table 1:** ‘Lost to contact after referral’ among patients referred from the Sihanouk Hospital Centre of Hope to peripheral health facilities for tuberculosis treatment, Phnom Penh, Cambodia (2008–2013)

Year	Tuberculosis patients diagnosed	Referred out for treatment initiation	Lost to contact after referral n (%)	Referred out for treatment continuation	Lost to contact after referral n (%)
2008	480	290	122 (42)	54	31 (57)
2009	557	327	102 (31)	47	35 (74)
2010	497	301	107 (36)	48	24 (50)
2011	465	240	45 (19)	68	19 (28)
2012	465	202	38 (19)	81	28 (36)
2013	440	223	150 (69)	67	34 (51)

From a patient perspective, not starting or completing TB treatment will negatively influence survival and from a public health perspective, infectious TB cases may transmit the disease to the community and hinder TB control. Such “missed cases” are now recognized as one of the most important challenges facing TB control globally [4].

We hypothesized that use of mobile phones to track referred TB patients might reduce the proportion of those being missed (LTCR). Importantly, since there is currently no nationally recommended mechanism to track patients referred out, the utility of this intervention will be of particular interest. Although there have been several mHealth studies that have assessed adherence after TB treatment has been initiated, none have assessed its use for retaining referred patients within the health system [5–8].

We aimed to assess if tracking of TB patients referred to peripheral health facilities using mobile phone technology can reduce LTCR. We thus determined the number and proportion of: 1) referred TB patients who could be contacted through a mobile phone and 2) LTCR among referred patients (for TB treatment initiation and continuation) after the introduction of mobile phone tracking.

## Methods

### Study design

A descriptive study involving follow-up of referred patients.

### Study population and period

All TB patients diagnosed in SHCH and referred to other peripheral health facilities for initiation or continuation of TB treatment during the period 1st May to 31st October, 2014 (intervention period) were included. The study was conducted between March and December 2014.

### Study setting

#### TB control strategy in Cambodia

The TB control efforts are led by the National TB Programme (NTP). As per WHO guidelines, Directly Observed treatment short-course (DOTS) strategy was introduced in public hospitals in 1994, and successfully expanded to achieve 100% DOTS coverage at health centre level in 2004. The National Centre for Tuberculosis and Leprosy Control (CENAT), under the Ministry of Health, coordinates TB activities in the country. The country has 25 provinces. For the purpose of health care provision, the provinces are further divided into 82 Operational Health Districts (OD). Each OD has its referral hospital and many peripheral health facilities (one per 10,000 population). Each OD has a TB supervisor who is responsible for supervising the Health Centres and reporting OD activities to the Provincial Health Department (PHD). At the PHD there is a medical TB supervisor and a laboratory supervisor. The TB supervisor is responsible for planning, coordination and supervision; he/she also receives quarterly TB reports from the ODs. Diagnosis and treatment services for TB patients are offered free-of-charge across the country.

#### The TB clinic of SHCH

All patients with symptoms suggestive of TB are offered sputum smear microscopy. Diagnosis of TB is based on WHO guidelines. In addition, those suspected to have drug resistant TB are tested for Rifampicin resistance using Xpert MTB/RIF and culture and drug susceptibility testing as per NTP guidelines [9]. TB patients residing in the vicinity of SHCH are initiated and continued on treatment at the TB clinic. Patients from other provinces are issued a referral slip along with their diagnosis details to initiate treatment in peripheral health facilities nearest to their homes. Very ill patients are admitted and started on TB treatment on an inpatient basis and once stable, are referred out to a peripheral health facility for treatment continuation. TB medication is given to the referred patients to cover for the transit period and avoid any treatment interruption. Patients are routinely requested to call back and inform the TB personnel at SHCH if they have reached the peripheral health facility and if they were started on treatment or not. Demographic, diagnostic and treatment details of every diagnosed TB patient are routinely collected in the data collection sheets and entered into the electronic TB database system of SHCH. Quarterly reports on case finding and treatment outcomes are submitted to NTP.

### Standard operating procedure for mobile phone tracking of referred patients

**Fig. 1:**
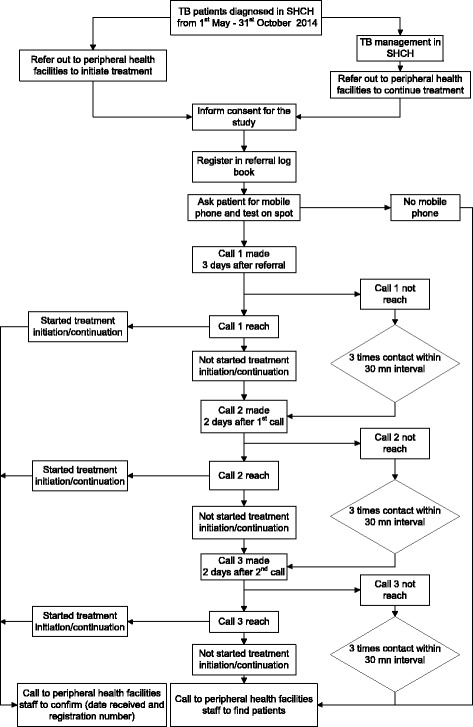
SOP for mobile phone tracking of referred patients from Sihanouk Hospital Centre of Hope to health facilities, Cambodia (May–October 2014)

Standard operating procedure for mobile phone tracking of referred patients is illustrated in Fig. 1. First, the functionality of the patient’s contact phone number was tested at the TB clinic in SHCH before referral by the health staff, following which a referral letter was provided. Three days after referral, TB staff in SHCH made a first call to the patient to check whether he/she had reached the designated peripheral health facility and was placed on TB treatment. If the patient expressed having problems with accessing the health facility or treatment services, guidance was provided and treatment services ensured in co-ordination with the staff of the peripheral health facility. In case the first call failed, a second and third call was made at an interval of two days. On each instance, three attempts were made to reach the patient within a time span of 30 min. An independent confirmation of treatment initiation was made by a dedicated person at the SHCH by calling the concerned health staff of the designated peripheral health facility. If the patient could not be reached after three attempts, the concerned health care provider of the peripheral health facility was contacted by telephone to actively trace the patient at home and initiate treatment.

### Data collection and analysis

Study participants were interviewed by a trained health care worker and data related to the study objectives were captured using a structured proforma. Data were double entered, validated and analysed using EpiData software (version 3.1 for entry and version 2.2.2.182 for analysis, EpiData Association, Odense, Denmark). Frequencies and proportions were calculated for categorical variables.

## Results

In 2014 (intervention period), a total of 193 TB patients were diagnosed of whom 109 patients were referred to peripheral health facilities – 92 for treatment initiation and 17 for continuation of treatment. Of the 109 referred patients, 107 (98%) had access to a mobile phone. The characteristics of these patients are shown in Table 2.

**Table 2:** Characteristics of tuberculosis (TB) patients referred out for TB treatment to peripheral health facilities from Sihanouk Hospital Centre of HOPE hospital (SHCH), Phnom Penh, Cambodia (May–October 2014)

Variable	N (%)
Total	109
Age	
< 34	22 (22)
35–55	47 (43)
> 55	40 (36)
Sex	
Male	55 (51)
Female	54 (49)
Occupation	
Farmer	31 (29)
Unemployed	29 (27)
Labourer	19 (17)
Small business	9 (8)
House wife	8 (7)
Other^a^	13 (12)
Co-morbidity	
No associated co-morbidity	87 (80)
HIV/AIDS	14 (13)
Diabetes mellitus	6 (5)
Other^b^	2 (2)
History of smoking	
Yes	35 (32)
No	74 (68)
History of alcohol intake	
Yes	39 (36)
No	70 (64)
Type of TB	
Smear positive Pulmonary TB	50 (46)
Smear negative Pulmonary TB	31 (28)
Extra pulmonary TB	28 (26)
Category of TB^c^	
First line treatment	88 (81)
First line retreatment	21 (19)
Type of referral	
Referred for treatment initiation	92 (84)
Referred for treatment continuation	17 (16)

^a^Driver (4), other (3), government employee (2), NGO worker (2), student (1) and soldier (1)

^b^Asthma (1), heart disease (1)

^c^First line treatment (newly diagnosed TB), First line retreatment (relapse, recurrent, failure)

About half of the patients were female with a median age of 51 years (interquartile range, 47–53 years). Most of them were ‘new’ patients with about one-fifth having associated co-morbidities including human immunodeficiency virus (HIV) and diabetes mellitus. The majority of the cohort included farmers, labourers and unemployed individuals. About one-third gave a history of tobacco smoking and alcohol consumption.

**Fig. 2:**
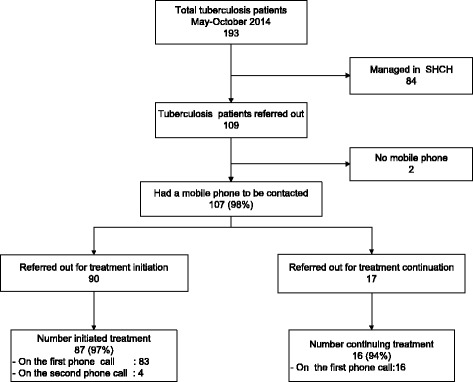
Patient flow of referrals to health facilities and a phone calls, Sihanouk Hospital Centre of HOPE, Cambodia (May–October2014)

Of the 107 patients who had access to a mobile phone, 103 (97%) could be contacted directly and were placed/continued on TB treatment. Figure 2 shows patient referrals to peripheral facilities, phone calls, and placement on TB treatment. Of six patients, five were eventually started on treatment through contacting the peripheral health facility (four could not be contacted personally through mobile phone and two did not have a mobile phone). Thus a total of 108 (99%) of 109 referred patients during the intervention period were eventually placed/continued on TB treatment at peripheral level.

## Discussion

This first study from Cambodia showed that all TB patients (except one) referred from a tertiary care hospital to peripheral health facilities for TB treatment could be tracked through a mobile phone. Compared to the previous years where 19–69% were being missed, this is a considerable improvement. Mobile phone tracking can thus serve as “a mechanism” to track referred patients and ensure treatment initiation. The findings may be applicable to other similar settings in LMIC [10].

The study strengths are that it was conducted within the framework of routine health service delivery and thus operationally relevant. Standard operating procedures for phone tracking were simple and feasible to implement, can be easily applied to other settings, and thus allows scale-up in Cambodia and beyond. Data were double-entered, validated and the staff involved in data collection were well trained - we thus believe the data are robust. This operational research study also responds to an identified national research priority.

There were some limitations. First, this was a descriptive study and we did not have an explicit control group selected using randomization. While comparison with a historical cohort showed that mobile phones might have helped in reducing LTCR, we cannot be conclusive about attributing the entire effect to mobile health intervention. Part of the difference might be due to different methods of data collection in the historical cohort which were passive and relied on information from patients calling back SHCH on their own initiative. So, some of the ‘missed’ patients in the historical cohort could easily mean ‘lack of documentation’ rather than ‘non-initiation of treatment’. Other differences could be due differential distribution of patient characteristics and other confounder variables in historical cohorts compared to study population. Another limitation is that we did not document the duration of calls and thus unable to cost this exercise. The average cost of mobile phone call is about 5 cents (United States Dollar) per minute, and it would seem logical to think that incurred charges might pale when mirrored against the public health and patient benefits of improved TB patient tracking. However, this is an area that merits further research. In the meantime, attempts should be made to reduce the overall cost of mobile phone calls when used for social programmes such as TB care. A way forward is to negotiate with phone companies to allow differential pricing of such calls or even ask for an offer of free calls as part of their corporate social responsibility. Finally, future research should also focus on qualitatively exploring the perspectives of health care providers and patients about the merits and challenges of this new intervention. Despite these limitations, it can safely be said that mobile phone tracking has a definite role in ensuring treatment initiation among referred TB patients and in improving documentation.

There are a number of study implications. First, given the significant retention observed among referred TB patients, this study provides preliminary evidence that using mobile phones might be of great value in TB care in high TB burden countries like Cambodia. We need better designed studies (preferably pragmatic randomized controlled trials based in programme settings) with a larger sample size to strengthen the evidence base.

Second, the introduction of mobile phone tracking was relatively easy to implement and encouragingly, a great majority of patients could be accessed through this device. Though, the resource implications (additional manpower needs, cost of calls) of such an intervention needs to be carefully worked out before making scale-up decisions.

Third, of six referred patients who could not be directly tracked through their own mobile phone devices, five could be traced through phone contact with peripheral health facilities and these individuals were eventually placed on TB treatment. Additional contact with health facility staff thus provided a “safety net” for ensuring patient retention. Ways forward to improve this net would include: a) developing and sharing a mobile phone directory of all health staff in the country and updating it on a periodic basis and b) adapting TB patient cards and TB registers to include phone numbers of patients and c) encouraging health staff to use hospital phones to actively seek feedback.

m-health is a cross-cutting intervention which has the potential for not only improving TB control but also for strengthening the health system as a whole. For example, m-health tracking could be extended to maternal and child health, non-communicable diseases and other forms of chronic care that involve referrals and follow up. Improvements in treatment adherence and bridging the provider-patient and provider-provider disconnect would be useful spin-offs [5, 11].

## Conclusion

In conclusion, this study provides preliminary evidence that using mobile phones was accompanied with a high retention among TB patients referred from a tertiary hospital setting in Cambodia. Future studies with better design and larger sample size are required to strengthen the evidence base before making national scale-up decisions.

## Abbreviations

CENAT: National Center for Tuberculosis and Leprosy Control
CI: Confidence interval
DOTS: Directly observed treatment short course
HIV: Human immunodeficiency virus
LMIC: Low and middle income countries
LTCR: Lost to contact after referral
mHealth: mobile health
NECHR: National Ethics Committee for Health Research
NTP: National tuberculosis program
OD: Operational health district
PHD: Provincial health department
SHCH: Sihanouk hospital center of HOPE
TB: Tuberculosis
WHO: World health organization
